# Gene Expression Analysis of Parthenogenetic Embryonic Development of the Pea Aphid, *Acyrthosiphon pisum*, Suggests That Aphid Parthenogenesis Evolved from Meiotic Oogenesis

**DOI:** 10.1371/journal.pone.0115099

**Published:** 2014-12-12

**Authors:** Dayalan G. Srinivasan, Ahmed Abdelhady, David L. Stern

**Affiliations:** 1 Howard Hughes Medical Institute and Department of Ecology and Evolutionary Biology, Guyot Hall, Princeton University, Princeton, NJ, 08544, United States of America; 2 Department of Biological Science, Rowan University, 201 Mullica Hill Rd, Glassboro, NJ, 08028, United States of America; China Agricultural University, China

## Abstract

Aphids exhibit a form of phenotypic plasticity, called polyphenism, in which genetically identical females reproduce sexually during one part of the life cycle and asexually (via parthenogenesis) during the remainder of the life cycle. The molecular basis for aphid parthenogenesis is unknown. Cytological observations of aphid parthenogenesis suggest that asexual oogenesis evolved either through a modification of meiosis or from a mitotic process. As a test of these alternatives, we assessed the expression levels and expression patterns of canonical meiotic recombination and germline genes in the sexual and asexual ovaries of the pea aphid, *Acyrthosiphon pisum*. We observed expression of all meiosis genes in similar patterns in asexual and sexual ovaries, with the exception that some genes encoding Argonaute-family members were not expressed in sexual ovaries. In addition, we observed that asexual aphid tissues accumulated unspliced transcripts of *Spo11*, whereas sexual aphid tissues accumulated primarily spliced transcripts. *In situ* hybridization revealed *Spo11* transcript in sexual germ cells and undetectable levels of *Spo11* transcript in asexual germ cells. We also found that an obligately asexual strain of pea aphid produced little spliced *Spo11* transcript. Together, these results suggest that parthenogenetic oogenesis evolved from a meiosis-like, and not a mitosis-like, process and that the aphid reproductive polyphenism may involve a modification of *Spo11* gene activity.

## Introduction

Polyphenisms are examples of extreme phenotypic plasticity, in which individuals of a species can develop into one of multiple discrete alternative phenotypes depending on environmental conditions [1]. Although polyphenisms exist in multiple taxa [2], the developmental, genetic, and molecular mechanisms that underlie polyphenisms remain poorly understood. Aphids exhibit a reproductive polyphenism that involves production of sexual or asexual females in response to environmental cues associated with seasonal changes. This polyphenism evolved approximately 200 million years ago and is found in most species of true aphids, adelgids and phylloxerans [3], [4]. The evolution of this trait and the mechanism by which aphids produce offspring asexually is not understood. Here, we examine the molecular and cellular basis for asexual reproduction in the pea aphid, *Acyrthosiphon pisum*.

The aphid reproductive polyphenism entails plasticity at the life history, cellular, and molecular levels. In the spring, an asexual female foundress hatches from an egg and gives live birth to clonal asexual daughters by parthenogenesis [5]. Parthenogenesis results in aphids that are genetically identical to each other and to their mother, aside from spontaneous mutations [6]. Asexual daughters continue parthenogenesis throughout the summer under long periods of daylight. In response to decreasing day length and cooler average temperatures in the fall, females produce – via parthenogenesis – sexual males and sexual females that can mate. Sexual females then lay overwintering eggs that hatch in the spring. Thus, aphids with the same genomes can produce drastically different reproductive forms.

**Figure 1:**
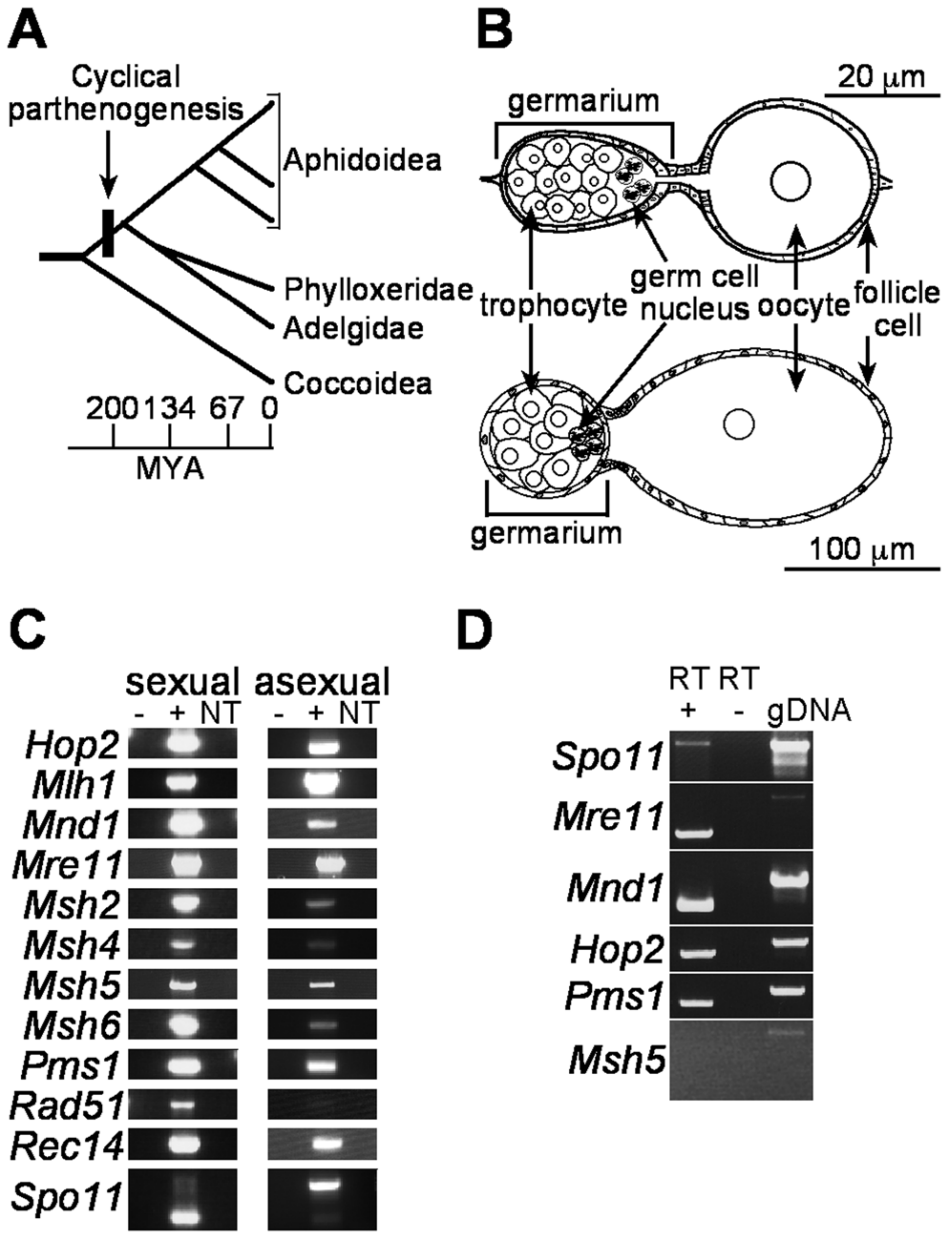
Meiosis genes are expressed in asexual aphids. *A.* Cyclical parthenogenesis among aphidomorph insects evolved from sexual ancestors as shown in this phylogeny (adapted from [3]). Time scale on bottom is in millions of years ago (MYA). *B.* Structures of an asexual ovariole (top) and a sexual ovariole (bottom) are shown. Ovarioles are oriented with anterior on the left. Scale bars are shown. *C.* Both sexual and asexual ovaries express meiosis genes. PCR products from cDNA template (+), control template lacking reverse transcriptase (RT) during cDNA synthesis (-), or from no template (NT) are shown. *D*. PCR of meiosis genes from the Tucson obligately asexual pea aphid strain. Lanes contain products from PCR using Tucson ovary cDNA template (+RT), template from cDNA synthesis reactions lacking RT (-RT), and genomic DNA (gDNA). Primers are the same as those used for *C*.

The major differences between asexual and sexual aphids are the structure of the ovaries and the fate of germ cells. Aphid ovaries consist of six to eight ovarioles. The anterior terminus of each ovariole consists of a germarium, which develops from germline precursor cells during embryogenesis into a syncytial collection of presumptive oocyte and trophocyte (nurse cell) nuclei enveloped by somatic follicle cells [7], [8] (Fig. 1B). From the germaria of both sexual and asexual ovaries, each presumptive oocyte nucleus, arrested in prophase, sequentially migrates out to form a follicle. The oocyte grows in size surrounded by the follicular epithelium.

In sexual organisms, oocytes are arrested in meiotic prophase, during which time double-strand DNA breaks are deliberately created throughout the genome to facilitate homologous recombination [9]. During meiotic prophase, homologous chromosomes pair, align, synapse and form crossovers [10]. Cytological and genetic evidence suggests that aphid sexual oocytes exhibit these hallmarks of meiotic recombination during development of the germarium and eventual release from the germarium [6], [7]. The aphid sexual oocyte arrests at metaphase I prior to fertilization. Upon fertilization, the sexual oocyte undergoes two rounds of nuclear division where homologous chromosomes (during the meiosis I reductional division) and then sister chromatids (during the meiosis II equational division) disjoin [7], [8], [11], [12]. Genetic data verify the occurrence of meiotic recombination and allelic segregation in sexual aphids [13].

In comparison to meiosis, little is known about the molecular mechanism of aphid parthenogenesis and asexual oocyte behavior. Genetic data and cytogenetic examination of aphid asexual oocyte formation and embryogenesis confirm that asexual lineages reproduce via clonal apomictic reproduction. Blackman [7] observed that homologous chromosomes appear to associate and pair in aphid asexual embryonic germ cells, but crossovers between homologs were not observed. However, the possibility of subsequent or brief formation of crossovers could not be ruled out. Soon after migrating out of the germarium, asexual oocyte nuclei divide only once, but no cell cycle arrest occurs during metaphase of this asexual oocyte division. This single division strongly resembles a meiosis II-like equational division: a short, asterless spindle apparatus forms and orients perpendicularly to the oocyte cortex; sister chromatids, instead of homologous chromosome pairs, segregate; and the oocyte undergoes an asymmetric cell division [7], [11], [14]. This division results in a one-cell embryo and in a polar body that does not appear to contribute to embryonic development [14]. The diploid one-cell asexual embryo then commences embryonic development as a syncytial blastoderm. As each oocyte sequentially departs from the germarium, the asexual ovariole eventually appears as a string of connected embryos at increasingly older stages. Genetic data from asexual mothers and daughters confirm the absence of crossovers, allelic segregation and male genetic contribution [13], though some recombination is limited to clusters of X-linked repetitive ribosomal DNA [13], [15]. Thus, it is not clear if the presence of partial homolog pairing, meiotic spindle formation and polar body extrusion indicates that parthenogenetic development evolved from a meiotic program, or whether these meiosis-like phenomena are coincident to the evolution of facultative asexuality in aphids.

To examine this question, we examined meiosis and germline gene activity in aphid ovaries. Much is known of the genetic requirements for meiotic recombination and meiotic division, and the sequences and functions of meiosis genes are conserved across fungi, plants, and animals [16]. Many meiosis genes retain ancestral functions in DNA damage repair. However, the meiotic recombination genes *Spo11*, *Hop2*, *Mnd1*, *Msh4*, and *Msh5* are required for, and appear to act solely during, meiotic recombination, based on genetic analysis in several model organisms. *Spo11* encodes a topoisomerase family member that creates double-strand DNA breaks during meiotic prophase that are critical for recombination and chromosome pairing in some species [17]. Additionally, independent of its role in recombination, Spo11 protein may be required for chromosome pairing early in prophase I [18]. Hop2 and Mnd1 proteins form dimers that facilitate meiotic homologous pairing and recombination [19]. Msh4 and Msh5 proteins are required for homologous chromosome pairing, recombination, and the control of crossover number [20]. The activities of meiosis genes must be tightly regulated to prevent compromise of genome integrity, although the expression of several meiosis-specific genes has been detected in somatic tissues [21]–[25].

We previously identified aphid orthologs of essential meiotic recombination genes (hereafter referred to as “meiosis genes”) [26]. All of these meiosis genes are found in single copies, indicating that the aphid reproductive polyphenism does not correlate with changes in meiosis-gene copy number, as it does in *Daphnia* [25]. Here, we found that both asexual and sexual morphs express all meiosis genes tested, including meiosis-specific genes. In contrast, we found that some genes of the Argonaute family, which are involved in suppressing transposon mobilization, are expressed solely in asexual ovaries. We also observed that *Spo11* transcript is spliced less in asexual aphids than in sexual aphids and that it is absent from asexual germaria. Together, these results suggest that aphid parthenogenetic embryogenesis evolved from a meiosis-like process.

## Results

### Meiosis-specific genes are expressed in both sexual and asexual pea aphid ovaries

Cytogenetic evidence indicates that facultatively asexual aphids produce progeny parthenogenetically by omitting meiotic recombination, crossover formation and meiosis I. To determine if these aspects of meiosis are modified or absent in asexual aphids at the molecular level, we assessed the expression of essential meiosis genes in sexual and asexual ovaries. All aphid meiosis genes we examined have complete gene models and, with the exceptions of *Spo11* and *Msh5*, are supported by multiple ESTs [26]. These EST libraries were derived from asexual pea aphids, which indicates that these genes are expressed in asexuals, at least at low levels [27], [28]. To confirm the expression of these genes in asexuals and to determine if they are expressed in germ tissue, we performed PCRs that spanned at least one intron of twelve meiotic recombination genes from cDNA template synthesized from third, fourth, and fifth instar sexual or asexual ovary total RNA. With this method, we detected transcripts for all meiosis genes from sexual and asexual ovaries but not from reactions without template (Fig. 1C). PCR on control cDNA synthesis reactions (lacking reverse transcriptase in order to assess genomic DNA contamination) did not amplify detectable product. With the exception of *Spo11*, which we discuss in detail below, all reactions yielded PCR products at or close to the predicted mRNA sizes. All of these products were cloned, sequenced and verified as representing spliced mRNA. This indicates that genomic DNA contamination did not contribute to the observed amplified PCR products. Asexual aphid ovaries appear to express genes that function solely in meiosis in other organisms (e.g., *Spo11*, *Hop2*, *Mnd1*, *Msh4* and *Msh5*), despite the absence of meiotic recombination in asexual aphids [13], [29], [30].

**Figure 2:**
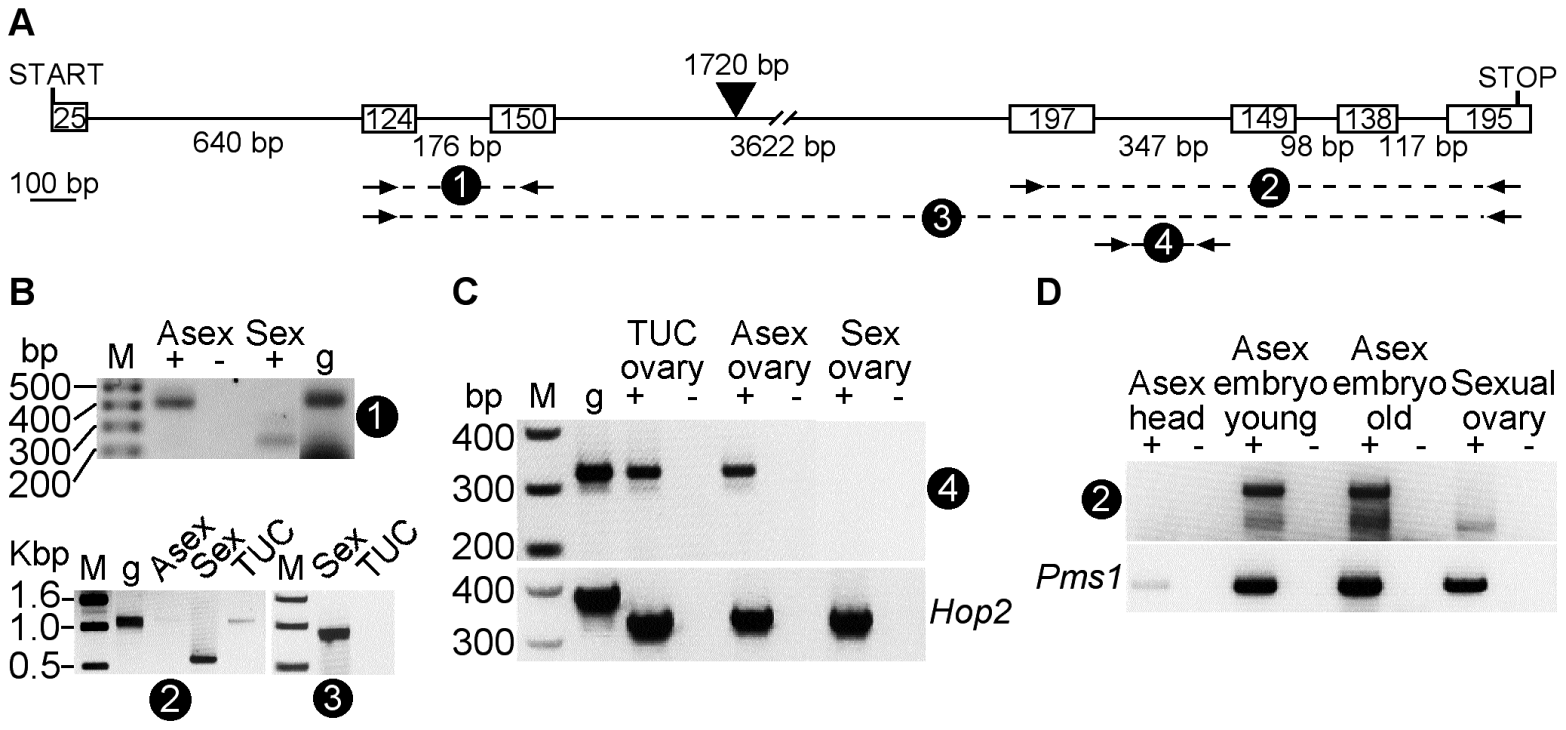
*Spo11* is spliced less in asexual aphids than in sexual aphids. *A.* Pea aphid *Spo11* gene structure is shown. Boxes refer to exons and lines refer to introns, with bp lengths indicated inside or below, respectively. The third intron includes a 1720 bp SINE2 retrotransposon, indicated by the arrowhead. PCR primer sets used in reactions shown in Panels *B*, *C* and *D* are indicated by a numbered dashed line and pair of arrows. The scale bar indicates 100 bp. *B.* Unspliced *Spo11* is detected in asexual aphids. Shown are PCRs from: genomic DNA (g); cDNA pools synthesized from RNA from sexual (“Sex”) LSR1.G1.AC ovaries, asexual (“Asex”) LSR1.G1.AC ovaries (+) and Tucson (“TUC”) pea aphid ovaries; and the RT-absent control template (-). Upper panel shows PCRs products from the second and third exons of *Spo11*. The bottom panel shows PCR products of the last four (*left)* or last six (*right*) exons of *Spo11*. DNA size standards are indicated on the marker (M) lanes of these gels. *C. Spo11* intron RNA is more abundant in asexually reproducing aphids than in sexual aphids. Shown are PCRs from: genomic DNA (g); cDNA pools synthesized from RNA from Tucson ovaries, asexual or sexual LSR1.G1.AC ovaries (+); or the RT-absent control template (-). Upper panel shows PCRs products from the fourth intron of *Spo11*. The bottom panel shows PCR products of full-length *Hop2* using the same template amounts. *D. Spo11* is expressed in asexual embryos *in utero*. Top panel shows *Spo11* PCR products using primer set 2, and the bottom panel shows *Pms1* PCR product. Pools of cDNA (+) or control template from cDNA synthesis reactions lacking RT (-) were created from the tissue sources listed above the lanes.

Our observations of *Spo11* expression do not fit a simple model of morph-specific gene expression. In our initial screen of meiosis genes, we observed a difference in the size and relative quantities of products in the *Spo11* PCR derived from sexual and asexual ovaries. The primers used in this PCR amplify sequence between and inclusive of exons 4–6 of *Spo11* (Fig. 2A). We sequenced the two major *Spo11* PCR products in Fig. 1C. The smaller product matched spliced mRNA, and the larger product matched genomic DNA (Fig. 2B, top panel). PCR reactions using other primer pairs (Fig. 2A) also detected *Spo11* transcripts corresponding to spliced and unspliced mRNA from sexual and asexual ovaries, respectively (Fig. 2B, bottom panels). Given that products were undetectable from control reactions lacking cDNA or template (Fig. 1C, Fig. 2B-D), it is likely that the larger PCR products reflect unspliced mRNA. This is supported by the relatively greater level of amplification of *Spo11* intron from asexual cDNA than from sexual cDNA (Fig. 2C). Taken together, these differences in the expression of *Spo11* transcript demonstrate a molecular difference between aphid sexual and asexual reproduction.

Cyclical parthenogenesis among aphidomorph insects evolved over 200 million years ago [4]. Subsequently, multiple strains of many aphid species have repeatedly evolved obligate asexuality and have lost the ability to produce sexual morphs [31]. The “Tucson” pea aphid strain is an obligately asexual strain and does not produce sexuals [32]. We wanted to test if the meiosis gene expression pattern seen in the cyclically parthenogenetic strain is still found in this obligately asexual strain. Using PCR of Tucson ovary cDNA, we detected expression of *Spo11*, *Hop2*, *Mnd1*, and *Pms1*; however, we observed little to no amplification of the meiotic recombination gene, *Msh5* (Fig. 1E). Amplification of the last four exons of *Spo11* from Tucson cDNA resulted only in unspliced cDNA (Fig. 2B, bottom left panel), and spliced Tucson *Spo11* cDNA was undetectable in PCRs of the majority of the locus using extension times that favor amplification of the spliced cDNA (Fig. 2B, bottom right panel). We were also able to amplify *Spo11* intron from Tucson cDNA (Fig. 2C). The lack of *Msh5* transcript and the lack of spliced *Spo11* transcript suggest that the meiotic program may be degenerating in this obligately asexual strain. Together, these results indicate that differences in *Spo11* expression and/or splicing correlate with the difference between sexual and asexual aphids.

### Meiosis gene transcripts localize in similar spatial and temporal patterns in sexual and asexual ovaries

Analysis of gene expression by PCR revealed expression of all meiosis genes in asexual females and in ovaries. However, dissected ovaries contain maternal germline and somatic tissue, as well as embryonic tissue. Thus, gene expression analysis by PCR could amplify transcripts present in any one of these tissue types. To localize the temporal and spatial patterns of meiosis gene expression, we first collected cDNA from three different dissected tissues: adult asexual heads, young asexual embryos, and old asexual embryos. We performed PCR of *Spo11* and *Pms1*, a conserved DNA mismatch repair gene ubiquitously expressed in humans [33]. We detected *Spo11* PCR products from young and old asexual embryo cDNA, whereas we detected *Pms1* PCR product in all tissues (Fig. 2D). This *Spo11* expression coincides with germarium formation and germ cell precursor divisions during embryonic development [14]; however, meiotic recombination or crossovers have not been observed during these asexual embryonic stages [7]. These results indicate that *Spo11* is transcribed during asexual embryonic development.

**Figure 3:**
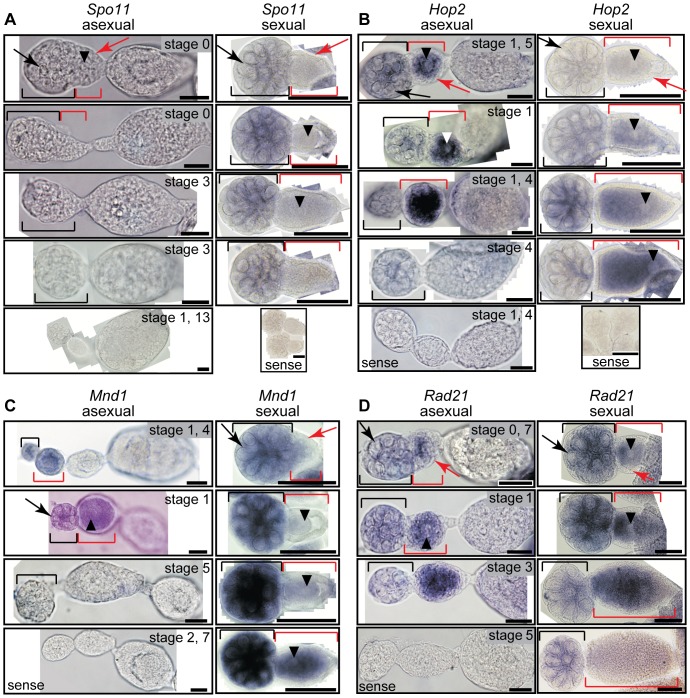
Meiosis genes are expressed in germaria and oocytes of both sexual and asexual aphids. *A. Spo11* staining. *B. Hop2* staining. *C. Mnd1* staining. *D. Rad21* staining. Black brackets indicate the germaria. Red brackets indicate the oocyte. Black arrows point to trophocyte nuclei in the germarium. Red arrows point to follicular epithelial cells. Black arrowheads point to the oocyte nucleus when in the plane of focus. Stages of asexual embryos in focus are labeled as described [14]. When two asexual embryos are observed in the same focal plane, both stages are indicated. Hybridizations using labeled sense probe for *Spo11* and for *Hop2* are shown. Black scale bars refer to 20 µm (asexuals) or 100 µm (sexuals).

We then surveyed the spatial pattern of expression of several meiosis genes in asexual and sexual ovaries with labeled antisense RNA probes. *Spo11* transcript was not detectable in the germline of asexual germaria and embryos despite using several different probes that included coding and/or intronic sequence (Fig. 3A, left). However, we observed *Spo11* transcript in sexual ovaries peaking in expression in early germaria and early oocytes and decreasing in expression in older oocytes (Fig. 3A, right). Transcript was not detected in the somatic follicular epithelium in either ovary type. These observations indicate *Spo11* is expressed at lower levels in the asexual germline than in sexual germline.

Other meiosis genes are expressed in similar patterns in asexual and sexual ovaries. We observed *Hop2* in both asexual ovarioles and sexual ovarioles (Fig. 3B). *Hop2* staining is present in asexual germaria and early oocytes, but it is absent from blastoderm stage embryos (Fig. 3B, left). *Hop2* transcript accumulates in sexual ovarioles as oocytes mature, and little transcript is detected in early oocytes (Fig. 3B, right). Similar to its dimerization partner *Hop2*, we observed *Mnd1* transcript signal in both asexual and sexual ovarioles, although *Mnd1* expression appears in sexual oocytes earlier than does *Hop2* expression (Fig. 3C). Transcripts of *Rad21*, which encodes a cohesin required for chromosome segregation and which should be expressed in both asexuals and sexuals, were observed in early oocytes and in germaria connected to early oocytes in both morphs (Fig. 3D). We observed no or little signal in somatic follicle cells or in somatic blastoderm embryonic tissues for these genes. Together, these results indicate that transcription of these meiosis genes is developmentally regulated in similar patterns in both sexual and asexual ovaries.

### Differential expression of germline genes between aphid morphs

**Figure 4:**
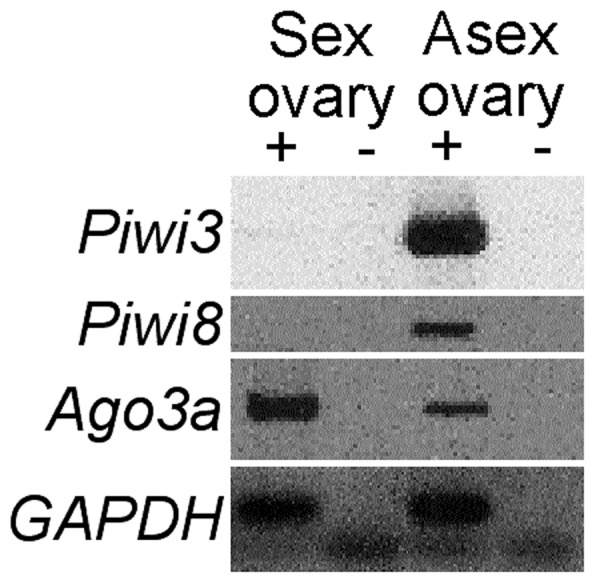
*Argonaute*-family genes are differentially expressed between aphid morphs. Shown are PCRs of aphid *Argonaute*-family genes *Piwi3*, *Piwi8* and *Ago3a*, along with GAPDH as a loading control. Products were amplified from cDNA pools synthesized with (+) or without (-) RT from RNA from asexual or sexual LSR1.G1.AC ovaries.

Phenotypic differences between asexual and sexual germ cells may involve genes that act primarily or exclusively in germline specification, maintenance and differentiation [34]. Among many genes that potentially act in this manner, we examined members of the *Argonaute* family, which encode proteins required for transposon suppression as well as transcriptional and post-transcriptional regulation of protein-coding gene expression in the germline and soma [35]. The *A. pisum* genome contains an expanded number of Argonaute family member genes relative to other insect clades: eight *Piwi* paralogs and two *Argonaute3* (*Ago3*) paralogs, all of which are lineage-specific and developmentally regulated [36]. We tested a subset of these genes for expression by PCR from cDNA pools made from asexual and sexual ovaries. We detected *Piwi3* and *Piwi8* transcripts amplified from asexual but not from sexual ovary cDNA (Fig. 4). Lu *et al*. [36] also observed expression in whole asexual aphids by qPCR, but they did not examine sexual aphid ovaries and did not detect *Piwi3* and *Piwi8* expression by *in situ* hybridization in either asexual germaria or asexual oocytes. Additionally, we detected *Argonaute3a* transcript in PCRs amplified from either sexual or asexual ovary cDNA (Fig. 4). Together, these data indicate that some Argonaute-family genes are differentially expressed in asexual and sexual germ cells.

## Discussion

Aphids evolved facultative reproductive plasticity from sexual ancestors over 200 million years ago [3]. Though the mechanisms regulating aphid parthenogenesis are unknown, the behavior of asexual oocytes is notable for the apparent lack of meiotic recombination, homologous crossovers, fertilization and meiosis I-like division prior to the single oocyte division [5]. One interpretation of these observations is that the meiotic program is turned off and that the asexual oocyte simply undergoes an asymmetric mitotic division to produce the one cell embryo. However, aspects of meiotic recombination and meiotic division appear to be intact in asexual oocytes, such as homologous chromosome pairing, meiosis-like division and polar body production [7], [8], [11]. If asexual oogenesis did not evolve from a meiosis-like process, then we would expect to observe the expression of few or no meiosis genes. Instead, we observed that asexual aphids express all meiosis genes tested and in patterns similar to those observed in sexual aphids. Specifically, genes involved in double-strand DNA break formation and homologous recombination, such as *Spo11*, *Hop2,* and *Mnd1*, are expressed in the asexual germline and early oocytes. Together, these results suggest that transcription of the meiosis program in the aphid germline may be constrained.

Since meiosis involves the deliberate production of DNA damage and genome recombination, the seemingly inappropriate expression of meiosis in asexual aphids could be deleterious and should be strictly regulated. Indeed, in mice and *S. cerevisiae, cis*-regulatory regions can restrict expression of meiotic genes to the germline [37]–[43]. In addition, studies from several model organisms identified multiple mechanisms restricting entry into meiosis at the transcriptional, posttranscriptional, translational, and posttranslational levels [44]–[46]. However, in aphids, we observed a conserved pattern of meiosis gene expression between morphs. The only exception we observed was for *Spo11*, a topoisomerase that creates double-strand DNA breaks that initiate recombination in meiosis. Aphid *Spo11* transcripts were detected in sexual nurse cells and oocytes, consistent with the *in situ* expression pattern of the *Drosophila Spo11* homolog, *mei-W68* [21]. However, *Spo11* is expressed in the asexual germ line below the level of detection by *in situ* hybridization. In addition, unspliced *Spo11* and apparent *Spo11* splicing intermediates are detected more readily in asexual ovaries than in sexual ovaries. Both observations suggest that less *Spo11* protein is available in asexual germ cells than in sexual germ cells, thereby preventing inappropriate recombination, DNA damage and chromosome pairing.

In addition to *Spo11*, other genes required for meiotic recombination and crossover formation are expressed in asexual aphids, yet these processes do not appear to occur. Low expression or inappropriate spatial or temporal expression could explain these expression patterns in part. However, our *in situ* results show that meiosis genes are expressed in the germline and not in somatic follicular or sheath cells. Expression of meiosis-specific genes outside of the meiosis context has been reported previously. Expression of *Drosophila mei-W68* was reported in *Drosophila* female soma and in males (which lack meiotic recombination) [21], and mutations in *mei-W68* can affect mitotic recombination rates [47]. *Spo11* expression was also detected in human and mouse somatic tissues [22]. Somatic expression of mammalian *Msh4* and *Msh5* has been observed [23], [24]. Therefore, while it remains unclear if these presumptive meiosis-specific genes have functions in asexual aphids, transcription of meiosis genes outside of the germ cell context and at low levels appears to be widespread in metazoans. Additional mechanisms may exist to limit the activity of meiosis genes to the appropriate context.

Regulated splicing of meiosis genes, especially *Spo11*, could serve, in part, as a mechanism to restrict meiotic initiation, homologous recombination and chromosome pairing to the appropriate times and tissues. For example, in fission and budding yeasts, several meiosis gene transcripts remain unspliced during vegetative growth, and these transcripts are spliced and presumably translated only upon transition to sporulation [48], [49]. Mouse somatic tissues express alternative transcripts of *Msh4* and *Msh5* that encode truncated and presumably nonfunctional proteins [23], [24]. Furthermore, several groups have reported, but not accounted for, observations of multiple transcripts of *Drosophila mei-W68* [21] and of alternative and regulated splicing of mouse and human *Spo11* transcripts [50]–[53]. Thus, alternative and regulated splicing of *Spo11* appears to be an underappreciated, widespread phenomenon among metazoans.

We hypothesize that developmentally regulated expression and splicing of aphid *Spo11* could serve to limit Spo11 protein levels and prevent meiotic recombination in asexuals. Since one purpose of meiotic recombination is to create interhomolog crossovers for proper orientation of homologous pairs during the meiosis I division [54], a decrease in *Spo11* protein would abrogate homolog disjunction during the asexual oocyte division. This implies the existence of either a factor that limits *Spo11* transcription and/or splicing in asexuals and the soma, or a germline-specific factor that promotes developmentally appropriate *Spo11* transcript splicing in sexual aphids. Alternatively, *Spo11* transcript and protein could be regulated differentially at the post-transcriptional, translational and posttranslational levels to prevent protein activity. To test this, we produced an antiserum directed to a peptide of *A. pisum* Spo11. However, this antiserum failed to detect aphid Spo11 by Western blotting and by immunofluorescent staining of both ovary types.

The similar patterns of meiosis gene expression in both sexual and asexual aphid ovaries imply that purifying selection has maintained the observed meiosis gene expression patterns in aphids. Lineages and species that lack sexual reproduction can provide a test of this hypothesis. The apparent loss of *Msh5* expression in the asexual Tucson strain supports a hypothesis that meiosis genes experience purifying selection in lineages that undergo sexual reproduction. Nevertheless, the Tucson strain still expresses most meiosis-specific genes. This may indicate that the Tucson pea aphid strain has only recently become obligately asexual and that an insufficient amount of time has elapsed to allow decay of meiosis genes. Alternatively, these genes may still be under purifying selection for non-meiotic functions in this strain. Examination of additional asexual pea aphid strains and other aphidomorphs will help to distinguish between these hypotheses.

While asexual and sexual aphid morphs appear to express a largely overlapping set of meiosis genes, aphid reproductive mode specification likely requires differential expression of other germline-specific genes. We tested the expression of several *Argonaute*-family genes, which are expressed in both germline and soma in metazoa and suppress transposons through piRNA-mediated transcriptional and epigenetic mechanisms [55], [56]. Honeybees, another polyphenic insect, exhibit temporal and caste-specific differences in *Argonaute* family member expression patterns during critical developmental periods [57]. We detected *Piwi3* and *Piwi8* expression from asexual ovaries primarily by PCR (Fig. 4), but neither transcript is detected in asexual germaria or oocytes by *in situ* hybridization [36]. Additionally, *Piwi2* and *Piwi6* paralogs are expressed specifically in the aphid asexual germline [36]. Thus, *Argonaute*-family gene expression patterns correlate with production of the aphid reproductive morphs. The individual roles of aphid *Argonaute* paralogs in the expression of the polyphenism, however, remain to be tested.

## Methods

### Aphid husbandry

LSR1.G1.AC and Tucson strains of pea aphid were reared on “Sutton dwarf” or “Windsor” *Vicia faba* broad bean plants (B&T Worldwide Seeds, Paguignan, France) kept at 19°C under long photoperiod regime (16 hours of light, 8 hours of darkness) to raise asexual aphids. Sexual aphids were induced by a “fall/winter” photoperiod (13 hours of light, 11 hours of darkness) at 16°C. Ovaries were dissected in cold PBS from third, fourth and fifth instar female asexual and sexual aphids.

### PCR

For RNA collection and cDNA synthesis, ten dissected ovaries or whole aphids were flash frozen in liquid nitrogen. Total RNA was extracted using the RNEasy Miniprep Kit (Qiagen) using a pestle and needle to disrupt and homogenize the tissue. Total RNA was treated with RQ1 DNase I (Promega) for 1 hour to remove genomic DNA, and the enzyme was inactivated for 10 min at 65°C in 1 mM EGTA. cDNA was synthesized from approximately 1 µg of DNase I-treated total RNA using the High Capacity cDNA Reverse Transcription Kit according to the manufacturer's protocol (Applied Biosystems) including reverse transcriptase (RT) or omitting RT (as a control template for PCR). cDNA synthesis reactions were diluted fiftyfold and used as template for PCRs of meiosis genes using GoTaq MasterMix (Promega) and forward and reverse primers at 500 nm each from 30 to 40 cycles. Portions of PCR reactions were electrophoresed in agarose, and imaged with ethidium bromide on a BioDocIt UV system (UVP). Gel images were scanned and cropped in Photoshop (Adobe). Scan moiré was reduced for some images using despeckle and unsharp mask filters in Photoshop. PCRs were cloned into TOPO-TA vector (Invitrogen), and inserts were sequenced. In the *Spo11* locus, a 1720 base pair (bp) SINE2 retrotransposon was identified in the third intron using RepeatMasker (www.repeatmasker.org).

Primers used for PCR are as follows:

Primer nameSequence

Spo11-F7 ATGATATTGGAAATGAAAGAACG

Spo11-R11 GACAAAAGGTAAATTATGATTGTGAAG

Spo11-F4 GGTGCAAAATCTTGGGAACTGGG

Spo11stop-R TTATTTTAGTGATACCTGTTGCAAAAGTTTATG

Spo11intronC-F GTAAAGCATTAAATTCTTAGTCC

Spo11intronC-R CTAAAACCAAAAAATATCTAATAGG

Hop2-F GCTTACTTGGTGACGGCTACGGC

Hop2-R CCAATATGTCCGTACACATGCG

Mlh1-F TGGCACTGGTATTCGCACTGAG

Mlh1-R CCAGGTAGGAGAAGCTGCACC

Mnd1-F TGCAGAGGAAAAACGTACCAGG

Mnd1-R TTCTTGCACCACGACTTCACTG

Mre11-F CCTCTTGCGTCATTATTGTTTGGG

Mre11-R CCTGGCTGGCAAATATAACCTG

Msh2-F GGACAGCAACCAAATGCTCACAC

Msh2-R CTTTTGCCGCCCATGTTTGG

Msh4-F TCCTTTACTAGACGTTGCTCGTACC

Msh4-R CCTTCTTCGCAAGACGTAGCTCG

Msh5-F CATGGATAATCTAGTTTGGCTCGG

Msh5-R GATGGTTTCCACAATGGAAGACAC

Msh6-F CTGCGGGTTCAAATGCAGG

Msh6-R CGCATGGATAACGTCCCTCG

Pms1-F CCGTCGGAAACGATCAAACTG

Pms2-R CCTTCACACGACCATTGCTGTC

Rad51-F GGGCATAAGTGAAGCAAAAGCTG

Rad51-R TGCGTCTCCAATTCCATCAGC

Rec14-F GCGATTGGGGTGAATTGCG

Rec14-R CATCTGACCATCATCTGCTCCAG

Hop2-qF AGAATCCGAGATTGAAGTGAAAACAC

Hop2-qR CATTTCGGCCACCTTGTCTT

Mnd1-qF AAGCATAGAAGTGGCCAAGGAA

Mnd1-qR CTTGCACCACGACTTCACTGA

Spo11-qF ACGGAGACACAATTCTATTCAAACATA

Spo11-qR AATGTAATACCCAGTTCCCAAGATT

Rad51-qF GTAGTTGCTCAAGTAGATGGTGCTT

Rad51-qR CATGAGCCATTATATTTCCACCAA

Rpn5-qF AGACTACAGTGCTACGTGCGATG

Rpn5-qR GCCAAACTTTCTGCGACAGC

GAPDH-F AAGTCAAGGAAGCCGCAGA

GAPDH-R ATTCCCGCCTTAGCGTCA

### *In situ* hybridization

Labeled sense and antisense RNA probes were synthesized with T7 or Sp6 polymerases from cloned full-length cDNA of *Spo11, Hop2, Mnd1*, and *Rad21* using the DIG RNA Labeling Kit (Roche). Probes were precipitated in sodium acetate and ethanol and resuspended in RNase-free water. Hybridizations were performed as described previously [58]. Dissected ovaries from ten female sexual or asexual aphids were permeabilized in heptane (5 min), fixed in 4% paraformaldehyde (20–30 min), washed in 50% methanol (30 min), and stored overnight at −20°C in 100% methanol. Methanol was replaced stepwise over several washes with phosphate buffered saline with 0.2% Tween-20 (PBST). Ovaries were postfixed in 4% paraformaldehyde for 20 min, washed in PBST, pretreated for 45 min in Detergent Solution (150 mM NaCl, 50 mM Tris HCl (pH 7.5), 1 mM EDTA, 1% SDS, 0.5% Tween-20), and washed extensively in PBST. Ovaries were then incubated in pre-hybridization buffer (1∶1 of PBST:SDS hybridization solution (50% formamide, 5x SSC, 0.1% Tween, 0.3% SDS, 50 ug/mL heparin, 100 ug/mL sonicated sperm DNA)) for 60 minutes and then in heat-denatured hybridization solution for 1 h at 65°C. Probes were added at a concentration of approximately 1–2 ng/µl, incubated for 16 h at 65°C, and ovaries were washed extensively in a dilution series of hybridization wash buffer (50% formamide, 5x SSC) in PBST. Ovaries were then washed in BBT (0.2% BSA in PBT), incubated with anti-DIG-AP antiserum at 1∶2000 overnight at 4°C, and washed extensively in BBT. Probe was detected by incubation of the ovaries in AP Reaction buffer (5 mM MgCl, 100 mM NaCl, 100 mM Tris pH 9.5, 0.2% Tween) and then incubated with NBT/BCIP (Roche) in the dark for 1–4 hours. Ovaries were then washed in PBS, and some ovaries were washed briefly (15 seconds) in 100% methanol to reduce background staining. Ovaries were then moved to 50% glycerol/PBS and mounted and sealed on slides. Hybridization experiments were repeated independently three times for each probe.
